# Speech in a consecutive series of children born with cleft lip and palate with and without syndromes and/or additional malformations

**DOI:** 10.1186/s12887-021-02783-0

**Published:** 2021-07-09

**Authors:** Kristina Klintö, Maria Sporre, Magnus Becker

**Affiliations:** 1 Department of Otorhinolaryngology, Skåne University Hospital, Malmö, Sweden; 2 Department of Clinical Sciences in Malmö, Lund University, Lund, Sweden; 3 Department of Plastic and Reconstructive Surgery, Skåne University Hospital, Malmö, Sweden

**Keywords:** Cleft lip and palate, Speech, Syndromes, Additional malformations

## Abstract

**Background:**

When evaluating speech in children with cleft palate with or without cleft lip (CP/L), children with known syndromes and/or additional malformations (CP/L+) are usually excluded. The aim of this study was to present speech outcome of a consecutive series of 5-year-olds born with CP/L, and to compare speech results of children with CP/L + and children with CP/L without known syndromes and/or additional malformations (CP/L-).

**Methods:**

One hundred 5-year-olds (20 with CP/L+; 80 with CP/L-) participated. All children were treated with primary palatal surgery in one stage with the same procedure for muscle reconstruction. Three independent judges performed phonetic transcriptions and rated perceived velopharyngeal competence from audio recordings. Based on phonetic transcriptions, percent consonants correct (PCC) and percent non-oral errors were investigated. Group comparisons were performed.

**Results:**

In the total group, mean PCC was 88.2 and mean percent non-oral errors 1.5. The group with bilateral cleft lip and palate (BCLP) had poorer results on both measures compared to groups with other cleft types. The average results of PCC and percent non-oral errors in the CP/L + group indicated somewhat poorer speech, but no significant differences were observed. In the CP/L + group, 25 % were judged as having incompetent velopharyngeal competence, compared to 15 % in the CP/L- group.

**Conclusions:**

The results indicated relatively good speech compared to speech of children with CP/L in previous studies. Speech was poorer in many children with more extensive clefts. No significant differences in speech outcomes were observed between CP/L + and CP/L- groups.

## Background

Research on the effect of cleft surgery on speech is still called for since we do not know what is the best timing or the best method for surgery [1–4]. When evaluating speech in children with cleft palate with or without cleft lip (CP/L), researchers usually exclude children with known syndromes and/or additional malformations (CP/L+) [2–4].

The reported incidence of cleft lip with or without cleft palate (CL/P) together with a syndrome and/or additional malformations is about 30 %, and it is about 45 % in cases of cleft palate without cleft lip (CP) [5–7]. Varying occurrence of CL/P and associated anomalies in different studies can be explained by differences in the methodology, for example different usage of definitions and inclusion/exclusion criteria, varying knowledge and availability of techniques to assess syndromes, and also differences in timeframes considered, population size, and participating population groups [5].

To be able to draw safe conclusions from studies on speech in individuals with CP/L, a stringent methodology must be used. Assessments should be performed from audio or video recordings, using a standardised speech stimulus and more than one listener to complete assessments [2–4, 8]. Also, results regarding the reliability of speech assessments need to be reported, and different types of clefts and ages should not be mixed [2–4, 8].

Few studies with adequate methodology for speech assessment have compared the speech of children with CP/L + and children with CP/L without known syndromes and/or additional malformations (CP/L-) at about 5 years of age. In a Swedish study with stringent methodology 5-year-olds with CP + at group level had poorer results on variables related to velopharyngeal function and articulation than peers with CP- [9]. Symptoms of Robin sequence are micrognatia, breathing difficulties and glossoptosis, and often a U-shaped cleft palate [10]. Studies comparing speech outcomes in patients with CP with and without Robin sequence have shown conflicting results, and there has been a lack of high-quality and long-term outcomes [10]. Hardwicke et al. [11] compared the speech of 24 5-year-olds with CP and Robin sequence and 24 peers with CP without Robin sequence matched for age, sex and cleft type, via consensus listening of recordings with standardised speech material. The reliability of speech assessments was not reported. The group with Robin sequence had nasality and cleft speech characteristics scores that were significantly higher than the group without Robin sequence.

We question if it is appropriate to exclude children with additional syndromes and/or malformations when publishing speech results after primary palatal surgery in children with CP/L. Therefore, the aim of this retrospective study was to present speech outcome of a consecutive series of 5-year-olds born with CP/L treated in a primary care university hospital, and to compare speech results of children with and without known syndromes and/or additional malformations.

## Materials and methods

### Participants

A total of 100 children participated, 20 with CP/L + and 80 with CP/L-. All 100 children were treated at Skåne University Hospital with primary palatal surgery in one stage with muscle reconstruction according to Sommerlad [12]. The children were divided into CP/L + and CP/L- groups based on information in medical records. The diagnosis of Robin sequence was made by the plastic surgeon and the criteria for diagnosis were micrognathia, glossoptosis and breathing difficulties. Children with at least one additional malformation, which required treatment or follow-up, were included in the CP/L + group. Furthermore, children that had been diagnosed with developmental disabilities and/or neuropsychiatric conditions that affected the child’s development were included in the CP/L + group. The children with CP/L- had been included in a previous study [13]. In the present study, speech outcomes of the children with CP/L + that had been excluded in the previous study [13] was investigated in relation to speech outcomes of the children with CP/L-.

### Palatal surgery

All included children were treated with intravelar veloplasty in one stage according to Sommerlad [12], combined with a modified technique by von Langenbeck [14]. In 96 out of 100 cases, the primary surgeon performed the surgery. In four cases in the CP/L- group, a second surgeon performed the surgery under supervision of the primary surgeon. Primary palatal surgery was performed between 8.8 and 19.1 months. In the CP/L + group, the mean age of surgery was 13.1 months (range 10.1–17.7), and in the CP/L- group 12.1 months (range 8.8–19.1). There was a significant difference in operating age between the groups (U = 506.5; p = 0.011), according to the Mann-Whitney U test.

### Documentation

The children’s speech was audio recorded in a recording studio at Skåne University Hospital, in connection with a routine follow-up at the age of 5 years (mean age 60 months; range 54–63 months). Speech was recorded using a Minidisc recorder (Sony MDS-302, Tokyo, Japan), a PC with the program Soundswell (Saven Hitech, Stockholm, Sweden), or a digital audio recorder (Zoom H4n, Hauppauge, NY, United States), together with a condenser microphone (Psytec Std61, Stockholm, Sweden; Sennheiser MD421-U-5, Wedemark, Germany; Red NT4, Sydney, Australia). The material recorded consisted of a single word test by picture naming, sentence repetition and continuous speech.

In 91 cases, the Swedish articulation and nasality test (SVANTE) was used for the elicitation of single words [15]. For eight children with CP/L-, the Scandcleft trials’ word test [16] was used and for one child with CP/L-, the word test in the randomised controlled trial Timing of Primary Surgery for Cleft Palate (TOPS) [17]. All word tests were designed according to the same principles, in order to analyse CP/L speech characteristics [16]. For one child with CP/L + only a few words were elicited. Since each word contained a target consonant, and it was required that at least half of the target consonants should be produced for the calculation of consonant outcomes included in the present study [15], this child was excluded in the analysis of consonant outcomes.

In addition, connected speech was recorded. A total of 90 children, 15 with CP/L + and 75 with CP/L- repeated the sentences from the SVANTE [15]. Each of the 13 sentences contained a recurring consonant, and the sentences were particularly sensitive to CP/L- related speech deviations. Furthermore, continuous speech was elicited for 12 children with CP/L + and 56 children with CP/L-. The continuous speech consisted of a conversation about a thematic picture [15], free spontaneous speech or retelling of the Bus Story [18, 19]. A total of 59 children (8 with CP/L + and 51 with CP/L-) both repeated sentences and produced continuous speech. For 31 children (7 with CP/L + and 24 with CP/L-), sentence repetition was the only connected speech recorded and for 9 children (four with CP/L + and 5 with CP/L-) free spontaneous speech.

### Editing

The recordings were transferred to a computer and de-identified. Two files were edited for each child; one consisting of single words from the word test, which contained the child’s speech followed by the test leader’s repetition of the word, and the second consisting of sentence repetition and/or continuous speech. Recordings from 28 children, seven with CP/L + and 21 with CP/L-, were randomly selected and duplicated for an intra-judge reliability assessment.

### Perceptual assessment and analysis of transcriptions

We wanted to investigate the same speech variables as used in the national cleft lip and palate (CLP) registry [20]. Three speech-language pathologists (SLPs) from three different CLP centres in Sweden assessed all audio recordings using headphones (Sony MDR-V700, Tokyo, Japan; Sennheiser HD 280 Pro, Wedemark, Germany; Sennheiser HD 205, Wedemark, Germany).

The SLPs transcribed target sounds from the single word tests using “semi-narrow” transcription (i.e. supplemental diacritics were used for characteristics common in CP/L speech), according to the International Phonetic Alphabet [21, 22]. Based on the entire speech material of each child, perceived velopharyngeal competence (VPC), i.e., an overall assessment of hypernasality, audible nasal air leakage, and weak articulation, was rated on a three-point scale with the scale values “competent/sufficient”, “marginally incompetent/insufficient” and “incompetent/insufficient” [15].

The main author performed analysis of phonetic transcriptions of target consonants in the single words, based on the first 59 words of SVANTE’s word test [15], or all words in the Scandcleft trials’ [16] and TOPS’ [17] word tests. Percent consonants correct (PCC) and percent non-oral errors were calculated for each child and SLP [15], by dividing the number of correct consonants and non-oral-errors with the total number of elicited target consonants. In PCC, the child’s production of the target consonant was scored as incorrect if the phonetic symbol differed from the target phonetic symbol. Age-appropriate errors were also scored as incorrect. Thus, varying types of lisp (such as inter-dental, lateral, supra-dental, postalveolar, retroflex, alveolo-palatal and palatal production of /s/) were scored as incorrect, along with weakening of /r/. Errors related to audible nasal air leakage and reduced pressure on consonants were not scored as errors in PCC. In calculation of non-oral errors, glottal and pharyngeal articulation and active nasal fricatives were scored as non-oral errors.

### Statistical analysis

Absolute agreement between and within judges of the overall scores of PCC and non-oral errors for each child, was calculated by the single measures intraclass correlation coefficient (ICC) with a 2-way intermixed model. Agreement of VPC was calculated using quadratic weighted Kappa. Descriptive data included measures of central tendency and distribution. Since the groups were small, and the results were not normally distributed, the Kruskall-Wallis test and Mann-Whitney U test was used for group comparisons. Differences in which *p* < 0.05 (two-tailed) were considered significant.

## Results

### Missing data

Over a 5-year period, 2005 to 2009, a total of 119 children were born with CP/L in the southern region of Sweden, 27 with CP/L + and 92 with CP/L-. Of the children with CP/L+, three were deceased, two had moved from the area, and two were unable to speak. Of the children with CP/L-, four had moved from the area, two dropped out from the follow-up at 5 years of age, and two had been treated with a different surgical method for palatal repair than the rest of the children. Further, the speech recordings failed for three children and one child was unwilling to participate in the speech assessment.

### Background data of participating children

Cleft type and gender distribution of participating children within the two groups are described in Table 1.

**Table 1:** Distribution of cleft type and gender in the group with cleft palate with or without cleft lip (CP/L) with syndromes and/or additional malformations (CP/L+) and the group with CP/L without syndromes and/or additional malformations (CP/L-)

Total number of children (*n* = 100)	CP/L+ (*n* = 20)			CP/L- (*n* = 80)		
	Girls	Boys	Total	Girls	Boys	Total
Cleft soft palate (*n* = 10)	1	1	2 (10 %)	2	6	8 (10 %)
Cleft soft and hard palate (*n* = 30)	2	6	8 (40 %)	12	10	22 (27.5 %)
Unilateral cleft lip and palate (*n* = 39)	2	4	6 (30 %)	8	25	33 (41.25 %)
Bilateral cleft lip and palate (*n* = 21)	2	2	4 (20 %)	4	13	17 (21.25 %)

For information regarding the occurrence of syndromes, additional malformations, developmental and neuropsychiatric conditions in the CP/L + group, see Table 2. Information regarding the type of syndromes and/or additional malformations in individual children cannot be provided to preserve confidentiality.

**Table 2:** The number of children with cleft palate with or without cleft lip with syndromes, additional malformations, developmental and/or neuropsychiatric conditions

Number of children (*n* = 20)	Syndromes, additional malformations, developmental disorders and/or neuropsychiatric conditions
4	Robin sequence only
2	Robin sequence and additional malformation/syndrome (organic heart disease, polydactyly, hypertelorism)
7	Other malformations, but no diagnosed syndrome (organic heart disease, hypospadias, vascular malformation, preauricular fibroma, ptosis, lip pits, lump foot, testis retention, hemifacial microsomia, lateral cleft lip)
4	Syndromes (van der Woude syndrome, Charge syndrome, Stickler’s syndrome)
3	Developmental disorders and/or neuropsychiatric conditions

In the CP/L + group, there were no postoperative fistulas. In the CP/L- group, six children had a postoperative fistula, and two underwent fistula closure before they were 5 years old. The other four children were not considered to require fistula closure. One child with CP/L + was treated with secondary speech-improving velopharyngeal flap surgery before the age of 5 years. In four children with CP/L+, assessment of the velopharyngeal function with video fluoroscopy was planned at the age of 5 years, or discussion was on-going regarding secondary surgery. Five children in the CP/L- group were treated with velopharyngeal flap surgery before the age of 5 years, and speech-improving surgery was planned for five children.

All children had met a SLP before the age of 5 years. In the CP/L + group the median number of visits was 18 (range: 6–51) and in the CP/L- group 12 (range: 2–50). During the period, the guidelines within the CL/P care program were changed, which meant that the children were called for fewer follow-up visits. Children born at the beginning of the time period were called to about 12 routine visits before the age of 5 years, and children born at the end of the time period to about five visits.

In the CP/L + group, two (10 %) of the children used hearing aids, and 12 (60 %) had or would begin treatment with ventilation tubes due to otitis media with effusion (OME). In the CP/L- group, no child used hearing aids and 34 (42.5 %) had or would begin treatment with ventilation tubes

### Reliability of speech assessments

Agreement between judges regarding speech variables was examined based on the recordings of all 100 children, and within the judges based on the recordings of 28 children. The results regarding agreement between assessments were interpreted according to Cicchetti [23]. For PCC, inter-judge agreement was excellent, with a single measures ICC value of 0.836 and a 95 % confidence interval (CI) of 0.758 to 0.889. Intra-judge agreement for PCC was also excellent, with ICC values varying between 0.967 and 0.981 and CIs from 0.931 to 0.991. For percent non-oral errors, inter-judge agreement was good, with a single measures ICC value of 0.735 and a CI of 0.655 to 0.804. Intra-judge agreement for percent non-oral errors was excellent, with ICC values varying between 0.945 and 0.986 and CIs from 0.89 to 0.993. Calculation with Kappa of inter-judge agreement of VPC was performed for two SLPs at a time. Inter-judge agreement of VPC was moderate to good (0.567; 0.568; 0.729). Intra-judge agreement of VPC was good to excellent (0.664; 0.666; 0.755).

### Outcomes of consonant production

Consonant production values were based on the mean of three SLPs assessments for each variable and child. In the total group, mean PCC was 88.2 and median 93.8 (Table 3). Mean percent non-oral errors for the total group was 1.5 and median 0. There were significant differences in results of consonant production related to cleft type (Tables 4 and 5), with poorer results in the group with bilateral CLP (BCLP) compared to groups with other cleft types (Table 5). The average measure of PCC was somewhat lower and the average measure of percent non-oral errors somewhat higher in the CP/L + group than in the CP/L- group, however, the differences were not significantly different (Table 3). The two children with cleft soft palate (SP)+, had medians of PCC and percent non-oral errors that was equal to the group with SP- (*n* = 8). In the group with cleft soft and hard palate (SHP)+ (*n* = 8), the median PCC was 87 and the median percent non-oral-errors 0, compared to 94.9 and 0 in the SHP- group (*n* = 22). In the group with unilateral CLP (UCLP)+ (*n* = 6), the median PCC was 91.4 and the median percent non-oral-errors 0, compared to 94.3 and 0 in the UCLP- group (*n* = 33). After excluding one child with BCLP + who only produced a few words in the single-word naming task, the median PCC in the BCLP + group (*n* = 3) was 84.7 and median percent non-oral errors 0.85, compared to 83 and 0.6 in the BCLP- group (*n* = 17).

**Table 3:** Results of percent consonants correct (PCC) and percent non-oral errors (Non-oral errors) in the total group (CP/L), the groups with (CP/L+) and without syndromes and/or additional malformations (CP/L-), and comparisons of results in the groups with CP/L+ and CP/L-

Outcome	CP/L (*n*=99)		CP/L+ (*n* = 19)		CP/L- (*n* = 80)		Mann-Whitney test	
	M	Md	M	Md	M	Md	U	*p*
	SD	min-max	SD	min-max	SD	min-max		
PCC	88.2	93.8	85	89	89	94.6	579.0	0.108
	12.8	35.1– 100	13.3	57.4–98.9	12.6	35.1–100		
Non-oral errors	1.5	0	2.3	0	1.4	0	704.5	0.548
	4.6	0 – 31.4	7.3	0–31.4	3.8	0–20

**Table 4:** Comparison of percent consonants correct (PCC) and percent non-oral errors (Non-oral errors) among the groups with cleft soft palate (SP), cleft soft and hard palate (SHP), unilateral cleft lip and cleft palate (UCLP) and bilateral cleft lip and palate (BCLP)

Outcome	SP (*n*=10)	SHP (*n*=30)	UCLP (*n*=39)	BCLP (*n*=20)	Kruskal Wallis test	
	Median	Median	Median	Median	Chi- Square	p
	Min-Max	Min-Max	Min- Max	Min-Max		
PCC	97.2	94.9	94.3	83	15.662	0.001**
	79.7 – 100	51.8 – 100	59.3 – 100	35.1 – 98.3		
Non-oral errors	0	0	0	0.6	15.816	0.001**
	0 –0.6	0 – 20	0 – 31.4	0 – 18		

**Significant at <.01

**Table 5:** Comparison of percent consonants correct (PCC) and percent non-oral errors (Non-oral errors) between total groups with cleft soft palate (SP; *n*=10), cleft soft and hard palate (SHP; *n*=30), unilateral cleft lip and palate (UCLP; *n*=39), and bilateral cleft lip and palate (BCLP; *n*=20) when analyzed pair wise (Mann Whitney test)

Groups	Outcomes			
	PCC		Non-oral errors	
	U	*P*	U	*p*
SP – SHP	121.0	0.364	136.0	0.565
SP – UCLP	122.5	0.072	192.0	0.912
SP – BCLP	27.5	0.001**	42.0	0.010*
SHP – UCLP	495.5	0.278	540.5	0.455
SHP – BCLP	144.5	0.002**	173.0	0.005**
UCLP – BCLP	210.0	0.004**	206.5	0.001**

*PCC* percent consonants correct, *Non-oral errors* percent non-oral errors

*/** Significant at <.05/<.01

Mean PCC for the 25 children with UCLP- who performed the SVANTE single word naming test was 91.16, median 96, SD 8.577. Mean PCC for the eight children with UCLP- who performed the Scandcleft word test was 91.38, median 92.5, SD 5.6. No significant difference was seen between groups (*p* = 0.640). Mean PCC for the 16 children with BCLP- who performed the SVANTE single word naming test was 79.81, median 83, SD 16.952. The child with BCLP- who performed the TOPS word test had a PCC score of 57.

### Perceived velopharyngeal competence

**Fig. 1:**
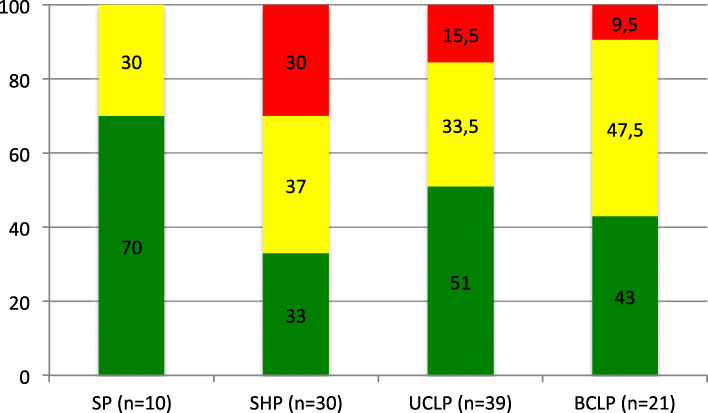
Perceived velopharyngeal competence (%) in the total groups of children with cleft soft palate (SP), cleft soft and hard palate (SHP), unilateral cleft lip and palate (UCLP) and bilateral cleft lip and palate (BCLP), where green refers to competent/sufficient, amber to marginally incompetent/insufficient and red to incompetent/insufficient

**Fig. 2:**
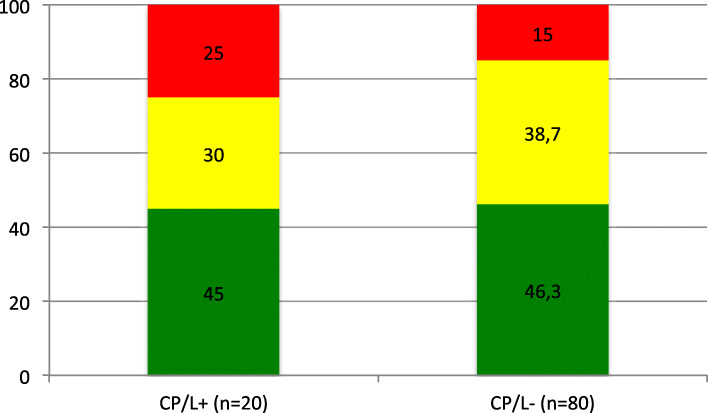
Perceived velopharyngeal competence (%) in the group with cleft palate with or without cleft lip (CP/L) with (CP/L+) and without syndromes and/or additional malformations (CP/L-), where green refers to competent/sufficient, amber to marginally incompetent/insufficient and red to incompetent/insufficient

VPC results were based on the median of three SLP assessments. Comparisons of VPC related to cleft type showed that the group with SP had the highest proportion of children with competent VPC and the group with SHP the lowest (Fig. 1). However, no significant difference was seen when Kruskall-Wallis test was performed (Chi- Square = 6.995; *p* = 0.86). The proportion of children with competent VPC was equivalent for the groups with CP/L+ (45 %) and CP/L- (46.3 %) (Fig. 2). A higher proportion of children within the CP/L + group (25 %) were determined to be incompetent with regard to VPC compared to children within the CP/L- group (15 %). No significant differences were observed between the two groups (U = -748.500; *p* = 0.63). In the SP + group and the BCLP + group no child was determined to have incompetent VPC. In the SHP + group four children (50 %) had incompetent VPC and in the UCLP + group one child (17 %).

## Discussion

The total group with BCLP had significantly poorer consonant production than groups with other cleft types (Table 5). These findings were expected and are in accordance with previous findings [13, 24]. No significant differences regarding the consonant variables were seen between the CP/L + group and the CP/L- group (Table 3). Due to the limited number of children no statistical comparisons based on cleft type were performed in the CP/L + group.

The mean values for PCC in the present study (CP/L + = 85 %; CP/L- = 89 %) were poorer than the mean value determined in the SVANTE norm data of 5-year-olds without CL/P (96.3 %) [15]. It should be considered that norm data were determined after children with speech and language disorders, hearing impairments, neurological or neuropsychiatric conditions, and anatomical/functional abnormalities in the oral cavity were excluded. This means that norm data were based on a population presenting no known speech difficulties. The results indicated relatively good speech compared to speech of children with CP/L in previous studies [4, 25]. Malmenholt et al. [25] examined PCC according to the same methods used in the present study, in 5-year-olds with CP/L + and CP/L-, aiming for a consecutive series of children. In their study, 39 % (*n* = 19) of the whole group (*n* = 52) had age-appropriate articulation proficiency, defined as a PCC score above − 1 SD, compared to 58.6 % (*n* = 58) in the present study. Thus, a higher proportion of children in the present study had age-appropriate articulatory proficiency than in the study by Malmenholt et al. [25]. In the study of Malmenholt et al. [25], a somewhat lower proportion of the total group (11.5 %) had BCLP and a somewhat higher proportion (23.1 %) had CP/L+, compared with the present study, where 16.2 % of the children with PCC scores had BCLP and about 19.2 % had CP/L+. Of the children with CP/L + in the study by Malmenholt et al. [25], 42 % had age-appropriate articulation proficiency, compared to 57.9 % in the present study.

There was no significant difference between groups regarding VPC. In line with previous findings [9], the group with SHP + had higher occurrence of symptoms related to incompetent velopharyngeal function than the SHP- group. In norm data, 2 % of the children were marginally incompetent with regard to VPC, and the remaining children were competent [15]. One child with CP/L + had undergone a secondary speech-improving surgery before the age of 5 years, and an assessment of velopharyngeal function with video fluoroscopy was planned for four children, prior to deciding whether they should undergo secondary surgery. In the CP/L- group, five children had previously undergone secondary speech-improving surgery and the surgery was planned for five. When children who had undergone secondary speech-improving surgery before 5 years of age were included in the category of children who were incompetent with regard to VPC, there still was no significant difference between the CP/L + group and the CP/L- group.

When interpreting the results, it should be taken into account that only children who fulfilled the criteria for the assessment were included in the analysis. One of the included children with CP/L + had not sufficiently developed spoken language for his/her consonant production results to be analysed. Two more children with CP/L + had not yet developed speech and were completely excluded from the study. Consequently, the children with CP/L + with the greatest communication problems had difficulties of such magnitude that their speech results could not be included.

All children in this study received the same surgical procedure for palate re-pair, between 8.8 and 19.1 months of age. The CP/L + group was operated on at a significantly older age (mean = 13.1 months) than the CP/L- group (mean = 12.1 months). The distribution of cleft types was uniform between both groups, and cleft type probably did not affect differences in operating age. Early palatal closure is considered to be better for speech than late palatal closure [1]. Although there was a significant difference between the two groups in timing of palatal closure, we do not know if this difference was clinically significant. When deciding on timing of palatal repair the child’s overall health condition must be taken into account. For example, surgery may have to be delayed in children with airway problems and cardiac anomalies [1]. This may explain why the mean age at surgery was higher in the CP/L + group than in the CP/L- group.

A higher proportion of children in the CP/L + group (60 %) than in the CP/L- group (34 %) were considered for ventilation tube treatment. The proportion of children undergoing ventilation tube treatment was slightly lower than rates reported in a study by Flynn et al. [26], but consistent with the results of a study, where children with CP + had a higher incidence of OME and poorer hearing than children with CP- [27]. As information regarding hearing was missing for the majority of the children in the present study, the speech results could not be studied in relation to hearing ability. It cannot be excluded that poorer speech results in some children were related to poorer hearing. Currently, audiometry is performed as a part of follow-up evaluations at 5 and 10 years of age at Skåne University Hospital, which means that for future studies, hearing data at these ages will be available.

Since the CP/L population is small, data must be collected for a long period in order to obtain a larger set of children with CP/L+ [5]. Studies of this magnitude demand a large quantity of resources and are difficult to implement, as data collection over a long period of time makes keeping other factors constant difficult. Issues such as timing and methods of operation, and methods and material used for data collection my change and affect comparisons [28]. In order to recruit participants for study within a reasonable time period, multicentre studies are warranted. Use of registry data will also make it easier to study speech in different subgroups within the CP/L population. This will be possible to perform in the future, since all six Swedish CL/P centres record speech data in the Swedish CLP registry at the same predetermined ages [20].

### Limitations of the study

The proportion of identified children with CP/L + was low compared to its previously reported incidence [5]. Only 25 % of the children with CP in the present study were part of the CP + group, compared to 45 % in a study by Cazolari et al. [6]. In the present study, the children with CP/L + were identified via medical record review. It is possible that an evaluation by a clinical geneticist had increased the proportion of identified children with CP/L+.

The speech material differed slightly among the children. Three different tests were used for elicitation of single words [15]. Furthermore, the materials used for elicitation of connected speech differed among the children. Although the word tests were designed according to the same principles [16], they differed regarding the number of target consonants, with a higher number of target consonants in SVANTE [15] than the two other tests [16, 17]. In addition, the proportion of /s/ as a target consonant was higher in the Scandcleft [16] and TOPS [17] word tests. When analysing the results, there were no indications that the difference in word tests used for analysis of consonant production affected the results.

VPC was rated on a three-point scale with the scale values “competent/sufficient”, “marginally incompetent/insufficient” and “incompetent/insufficient” [15]. At the time when the perceptual assessment in this study was performed there were no further guidelines on how to perform the rating. Later on, we have elaborated the national guidelines on how to use this scale.

The number of participants in the total group and also the CP/L + group were low. In order to make statistical comparisons between children with CP/L + and CP/L- that had different cleft types and compare speech results of subgroups of children with specific additional conditions, a considerably larger number of children should have been included in the study.

## Conclusions

The group with BCLP had poorer results of consonant production than groups with other cleft types. No significant differences in speech outcomes were observed between CP/L + and CP/L- groups. Thus, the results indicated that it may not be motivated to exclude children with CP/L + when evaluating CP/L speech. However, the number of participants in the total group and also the CP/L + group were low, and speech in subgroups related to cleft type and additional syndromes and/or malformations could not be statistically analysed. Studies using larger groups of children with CP/L + and CP/L- are warranted. Inter-centre and registry studies, which provide data for increased numbers of participants, will facilitate the investigation of speech in sub-groups of children with CP/L+.

## Data Availability

The dataset analysed during the current study is available from the corresponding author to researchers on reasonable request.

## Abbreviations

BCLP: Bilateral cleft lip and palate
CI: Confidence interval
CLP: Cleft lip and palate
CL/P: Cleft lip with or without cleft palate
CP: Cleft palate without cleft lip
CP/L: Cleft palate with or without cleft lip
ICC: Intraclass correlation coefficient
OME: Otitis media with effusion
PCC: Percent consonants correct
SHP: Cleft soft and hard palate
SLP: Speech-language pathologist
SP: Cleft soft palate
SVANTE: The Swedish articulation and nasality test
TOPS: Timing of Primary Surgery for Cleft Palate
UCLP: Unilateral cleft lip and palate
VPC: Perceived velopharyngeal competence
+: With syndromes and/or additional malformations
-: Without known syndromes and/or additional malformations
